# Diketopyrrolopyrrole Based Organic Semiconductor Materials for Field-Effect Transistors

**DOI:** 10.3389/fchem.2021.671294

**Published:** 2021-04-14

**Authors:** Xiangyu Zou, Shuaiwei Cui, Junqiang Li, Xueling Wei, Meng Zheng

**Affiliations:** ^1^National and Local Joint Engineering Laboratory for Slag Comprehensive Utilization and Environmental Technology, School of Materials Science and Engineering, Shaanxi University of Technology (SNUT), Hanzhong, China; ^2^Key Laboratory of Rubber–Plastic of Ministry of Education (QUST), School of Polymer Science and Engineering, Qingdao University of Science and Technology, Qingdao, China; ^3^Qingdao Haiwan Science and Technology Industry Research Institute Co., Ltd., Qingdao, China

**Keywords:** DPP, organic semiconductor materials, organic field effect transistors, D-A typed materials, molecular design concept

## Abstract

Over the past several decades, organic conjugated materials as semiconductors in organic field effect transistors (OFETs) have attracted more and more attention from the scientific community due to their intriguing properties of mechanical flexibility and solution processability. However, the device fabrication technique, design, and synthesis of novel organic semiconductor materials with high charge carrier mobility is crucial for the development of high-performance OFETs. In the past few years, more and more novel materials were designed and tested in the OFETs. Among which, diketopyrrolopyrrole (DPP) and its derivatives, as the electron acceptors to build donor-acceptor (D-A) typed materials, are the perspective. In this article, recently reported molecules regarding the DPP and its derivatives for OFETs application are reviewed. In addition, the relationship between the chemical structures and the performance of the device are discussed. Furthermore, an outlook of DPP-based materials in OFETs with a future design concept and the development trend are provided.

## Introduction

During the past decade, organic semiconducting materials including π-conjugated small molecules and polymers have attracted increasing attention due to their potential applications in organic electronic devices such as organic field-effect transistors (OFETs) and organic photovoltaics (OPVs) (Zhao et al., 2016; Huang et al., 2017). Compared to traditional Si- and GaAs-based technologies, organic semiconductors offer unique features, such as their mechanical flexibility, variable optical band gaps, and low-temperature large-area solution processability. The transistor is the fundamental building block of modern electronic devices and is used to amplify and switch electronic signals (Guo X. G. et al., 2014). To fabricate high-performance OFETs, charge transfer mobility (*μ*), threshold voltage, and current on/off ration are the three key factors. Among these three factors, high charge carrier mobility is the most important and a challenging, since the existing organic semiconductor's charge transfer mobilities are far behind compared to the inorganic Si-based ones, resulting in it not meeting the commercial demand (Qiu et al., 2020). Except the device fabrication technique, the chemical structures of the organic semiconductors play a key role for improvement of the charge carrier's mobility. This could be ascribed that in the OFETs, charge carriers need to be frequently transferred between individual molecules and within molecules in order to transport from one electrode the another (Zhang et al., 2017a, 2020). The chemical structures of the organic materials are easily tunable with diverse core/backbone architectures, which can fulfill better solid-state packing, thin-film morphology, overlap of the molecules, π-π stacking and the crystalline, resulting in improvement to the inter-charge transfer mobility. In addition, novel organic molecules with large π-conjugation system, good planarity, and strong donor-acceptor groups are beneficial for the intra-charge transfer (Guo X. G. et al., 2014).

The charge is divided into electron and hole, based on whether the OFETs are distinguished into p-type (hole transfer type), n-type (electron transfer type), and ambipolar type (electron and hole transfer type). Currentlys, the n-type and ambipolar type OFETs have received more attention from the scientific society, because the existing highest electron mobility and the stability of the OFETs are much lower than the reported highest hole mobility. Research revealed that the design and synthesis of novel organic semiconductor materials with high charge carrier mobility is crucial for the development of high-performance OFETs. In the past few years, more and more novel materials were designed and tested in the OFETs. Among which, diketopyrrolopyrrole (DPP) and their derivatives, as the electron acceptors to build donor-acceptor typed materials, are the perspective (Donaghey et al., 2013; Lin et al., 2018; Lee et al., 2019). In this article, recently reported molecules regarding to the DPP for OFETs application are reviewed. In addition, the relationship between the chemical structures and the performance of the device are also discussed. Finally, we provide our perspective to address the existing critical points of high-performance OFETs and the molecular design concept regarding the DPP derivatives, by outlining potential evolution trends. We hope that this work will promote new insights and further research studies to boost the development of OFETs for the next-generation applications.

## DPP-Based Conjugated Materials

DPP-based molecules were often constructed by a core containing two amine units and carbonyl groups with bicyclo, and end flanked by the aromatic groups. The core endowed the DPP derivatives with strong electron deficiency properties, which can be used to build donor-acceptor system molecules. In addition, once the aromatic groups were flanked with small units, the conjugated backbone of the DPP exhibited highly planar which endowed the high intra-charge transfer mobility (Bao et al., 2020). DPP-based materials have often exhibited extraordinarily strong π-π interaction and aggregation properties between the neighboring DPP moieties, resulting in the materials having beneficial properties for inter-charge transfer mobility. Hence DPP and its derivatives-based materials were widely studied and used in build high-performance OFETs (Qu and Tian, 2012).

### DPP

**Figure 1:**
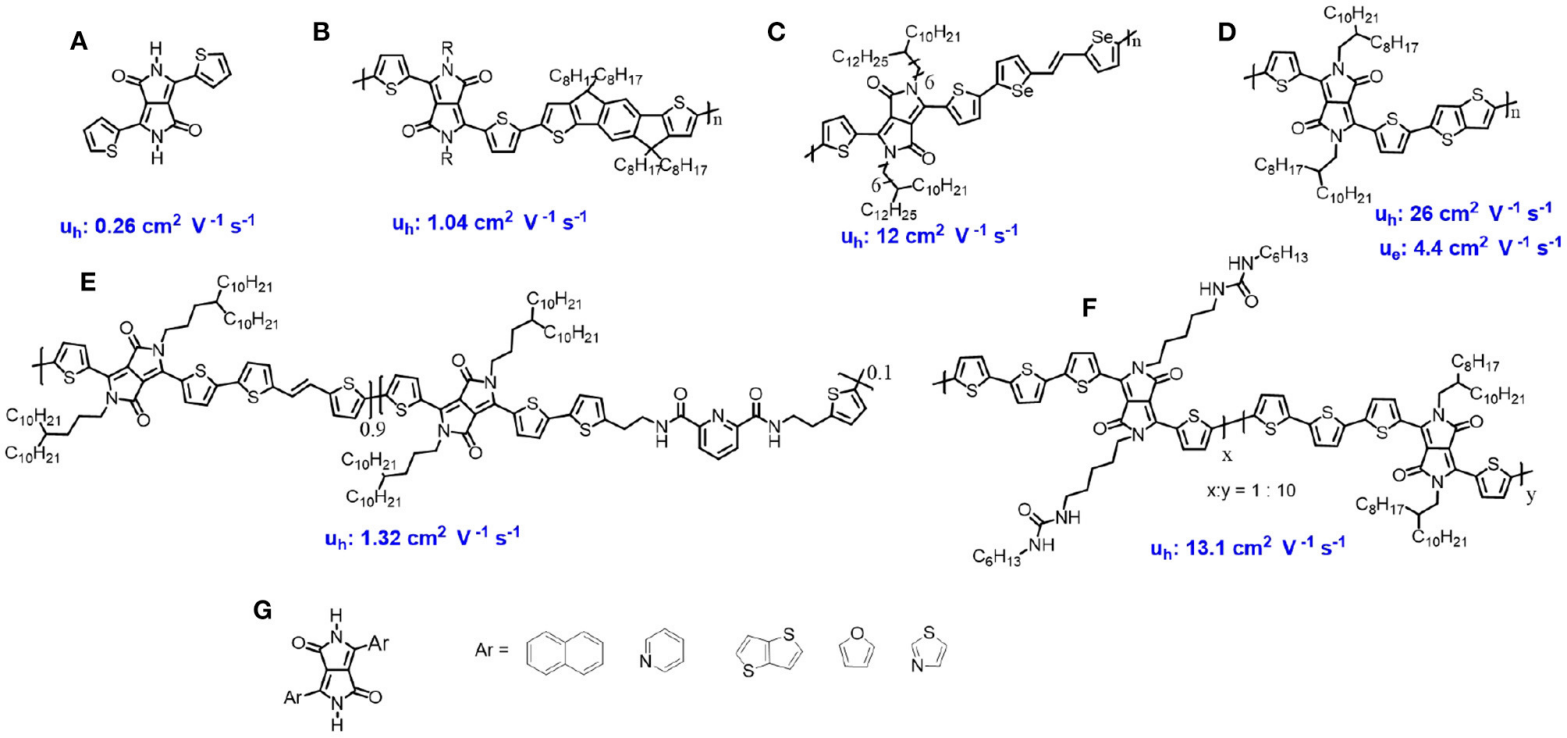
Chemical structures of DPP-based materials for OFET and their charge transfer mobility.

Since the first DPP-based materials application in OFETs were reported by Bürgi et al. with a hole mobility (*μ*_h_) of 0.1 cm^2^ V^−1^ s^−1^ and electron mobility (*μ*_e_) of 0.09 cm^2^ V^−1^ s^−1^ (Lukas et al., 2008), the DPP derivatives received more and more attention from chemists as one of the most promising building blocks in organic semiconductors. Recently, Zhang's group reported the hydrogen bonded DPP based small molecules with a hole mobility of 0.26 cm^2^ V^−1^ s^−1^ (Zhang et al., 2017a) and the polymer with a hole mobility up to 1.02 cm^2^ V^−1^ s^−1^ (Zhang et al., 2019) (Figures 1A,B). Kang et al. using DPP as the acceptor to synthesize D-A type polymers with the *μ*_h_ up to 12 cm^2^ V^−1^ s^−1^ (Kang et al., 2013) (Figure 1C). Until now, the best DPP-based OFET was reported by Luo et al. Through intruding tetramethylammonium iodide into the DPP polymer, good ordered lamellar packing of the alkyl side chains and inter-chain π-π interactions film were formed, meanwhile the *μ*_h_ was up to 26 cm^2^ V^−1^ s^−1^ and *μ*_e_ up to 4.4 cm^2^ V^−1^ s^−1^ (Luo et al., 2016) (Figure 1D). This work reveals that the DPP chromophore is a potential building blocks in the semiconductors materials.

In 2016, Bao's group introduced 2,6-pyridine dicarboxamide with non-conjugated alkyl units into the DPP-based polymer backbone, with the interaction between the C=O group and N-H group in the adjacent molecules, which could form hydrogen bonding between the neighboring molecules. These kinds of polymers showed not only high hole transfer mobility (1.32 cm^2^ V^−1^ s^−1^), but also exhibited self-healable properties (Oh et al., 2016) (Figure 1E). Almost at the same time, Yao and co-authors, introducing urea-containing alkyl chains into the DPP based polymers, developed hydrogen bonded polymers with a hole mobility up to 13.1 cm^2^ V^−1^ s^−1^ (Yao et al., 2015) (Figure 1F). These works indicate that hydrogen bonding association could significantly improve the DPP-based charge transfer mobility.

The flanked aromatic group attached to the DPP core are crucial for the design DPP-based molecules. The most reported flanked units are the thiophene units. Besides that, other groups were also introduced as the flanked units attached to the DPP core to build novel chromophore, such as the naphthalene (Liu et al., 2018), furanyl (Sonar et al., 2012), thiazole (Li et al., 2014), pyrrinde (Li et al., 2017), thienothiophene (Jiang et al., 2017), and so on (Figure 1G). These new materials also showed good performance in the OFETs.

### isoDPP

**Figure 2:**
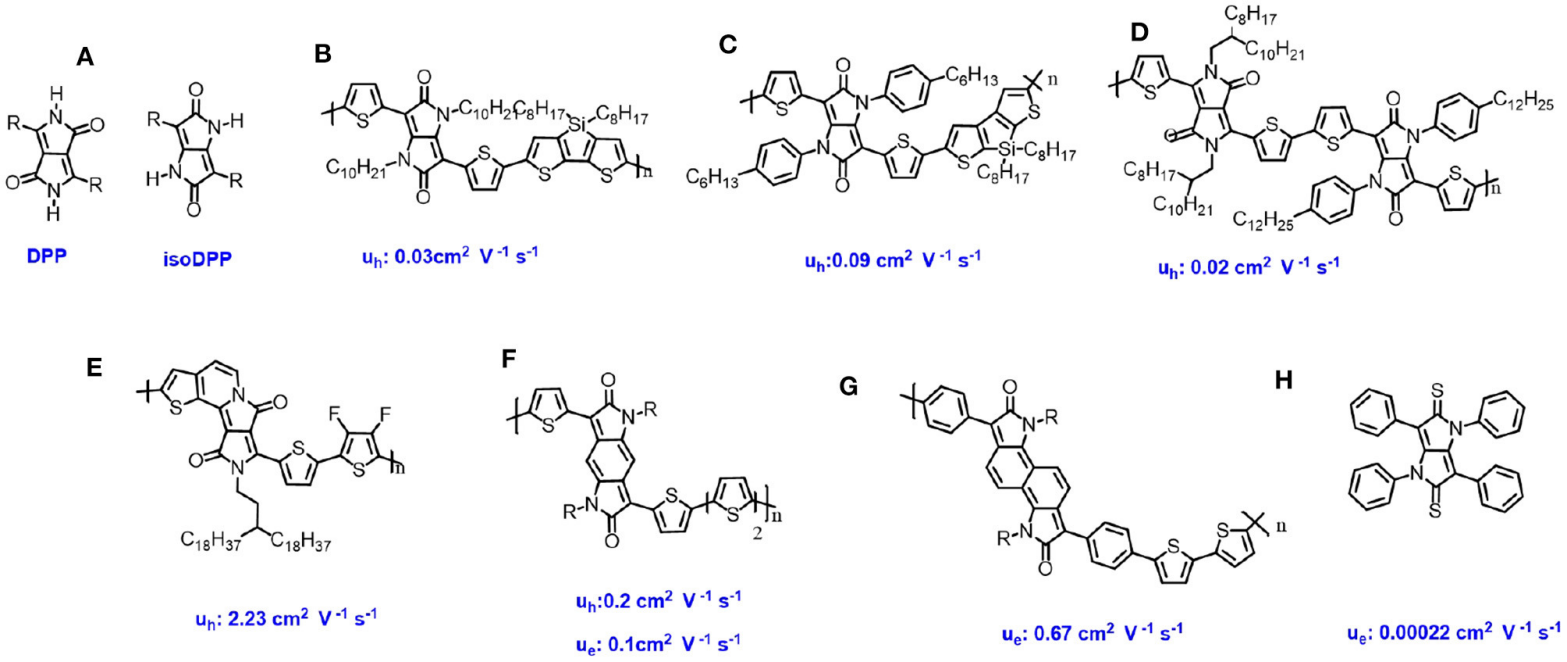
Chemical structures of isoDPP- and DPP based derivatives for OFET and their charge transfer mobility.

IsoDPP is a regioisomer of DPP, the chemical structure of which is similar to the DPP with a switching position of the carbonyl group and the nitrogen atom (Figure 2A). Compared to the DPP-based semiconductors, the isoDPP-based one received less attention, though it has as much potential as the DPP-based ones. IsoDPP was firstly developed roughly 30 years ago in one step from pluvinic acid (Rochat et al., 1985; Deng et al., 2019). The first isoDPP-based polymers were synthesized by Tieke's group in 2011 (Welterlich et al., 2012), while the first isoDPP-based polymer in OFETs were tested by Facchetti's group in 2013 (Lu et al., 2013). The polymers were constructed by the donor-acceptor system, isoDPP units as the acceptor and the dithieno[3,2-b:2′,3′-d]silole units as the donor, which showed p-type behavior with a hole mobility of 0.03 cm^2^ V^−1^ s^−1^ (Figure 2B). In 2018, Zhang et al. reported similar polymers with the same polymer backbone but different alkyl-chain. This polymer showed a hole mobility of 0.09 (Zhang et al., 2018) (Figure 2C). The high hole mobility could be ascribed to the high molecular weight, which is beneficial for the charge transfer within the single polymer backbone. In addition, the author reported that through one-step thionation reaction using Lawesson's reagent, oxygen–sulfur exchange in the isoDPP core happened, resulting in a new chromophore isoDTPP. The isoDTPP based polymers showed hole mobility up to 0.49 cm^2^ V ^−1^ s ^−1^, which is almost five times larger compared to the isoDPP-based one, and the electron mobility to 0.29 cm^2^ V ^−1^ s ^−1^. This indicates that the isoDTPP is a promising building block for high performance organic semiconductors^1^. Recently, Guo et al. reported a copolymer containing isoDPP and DPP in the polymer backbone to construct acceptor-acceptor system polymers (Guo X. et al., 2014) (Figure 2D). This polymer showed ambipolar behavior with balanced holes and electrons mobilities around 0.02 cm^2^ V^−1^ s^−1^. The charge carrier mobility of this polymer exhibited around two to three orders of magnitude higher than the reported DPP-based “homo” -polymer (Zoombelt et al., 2010). This could be ascribed to the fact that the isoDPP polymers showed good order and quite close packing distance of 0.38 nm in the solid state, which is beneficial for the charge carrier transport between the neighboring molecules.

Not like the DPP-based materials, the isoDPP-based materials were only designed by two types, such as phenyl-flanked and the thiophene-flanked isoDPP. Gendron et al. (2014) and Zhang et al. (2013) prepared the single crystal of these two different small molecules, which showed that the core of isoDPP was fully coplanar as DPP. The studies showed that using a thiophene ring instead of a phenyl ring attached the isoDPP core could significantly improve the planarity of the conjugated isoDPP backbone. In addition, the thiophene units exhibits stronger donor units than the benzene groups, which could form the donor-acceptor system between the thiophene and the isoDPP core. A high planar conjugation backbone and stronger donor-acceptor system is beneficial for the intra-charge transfer, thus the thiophene-flanked isoDPP has more potential. IsoDPP chromophore as a novel building block has potential in semiconductors materials, though it has not received the desired attention. Future research regarding to the isoDPP-based materials should be focused on adjusting the flanked groups (Zhao et al., 2019), improving the planarity materials conjugated backbone (Zhang et al., 2016; Li et al., 2021), introducing functions units into the core (Oh et al., 2016), and so on.

### DPP-Based Derivatives

In the past few years, various kinds of DPP-based derivatives were widely developed and used as the organic semiconductors in OFETs. In 2020, Shi et al. designed a novel building block, denoted as a half-fused DPP, in which one of the flanking thiophene units is fused to the DPP core *via* a carbon-carbon double bond at the N-position (Shi et al., 2020). In this work, through Stille coupling, donor-acceptor polymer, the difluorothiophene as donor, and the half-fused DPP as acceptor, was synthesized with a molecular number weight of 24.4 kDa (Figure 2E). The obtained polymer exhibited ambipolar behavior with *μ*_h_ of 2.23 and *μ*_e_ of 1.08 cm^2^ V^−1^ s^−1^, while the non-fused DPP based polymer with similar chemical structures showed quite low charge carrier mobility with *μ*_h_ of 0.78 and *μ*_e_ of 0.24 cm^2^ V^−1^ s^−1^. The significant improvement charge carrier mobility should be ascribed that: the high planar backbone, monosubstituted alkyl chain, instead disubstituted alkyl chains reducing the steric crowding, a short π-π stacking distance and lower LUMO energy levels. This research reversed that the planarity of the polymer backbone is crucial for high performance organic semiconductors.

Enlarging the π-conjugation system of isoDPP core results in benzodipyrrolidone (BDP) and naphtodipyrrolidone (NDP). Compared to the isoDPP, the BDP, and NDP showed similar chemical structures except the core are tri- and tera-cyclic (Deng et al., 2019). Rumer et al. reported BDP based polymers with ambipolar behavior with μ_h_ and μ_e_ of 0.2 and 0.1 cm^2^ V^−1^ s^−1^, respectively (Rumer et al., 2013) (Figure 2F), while a similar polymer consisting of NDP based polymer exhibited n-type behavior with μ_e_ of 0.67 cm^2^ V^−1^ s^−1^ (Zhang et al., 2017b) (Figure 2G). The different properties of these semiconductors could be due to the fact that the π-conjugation extension could not only enlarge the charge transfer pathway, but also adjust the frontier molecular orbitals.

Upon thiolation reaction, the carbonyl of the DPP core could be easily transferred into thiocarbonyl resulting DTPP (Gendron et al., 2017). The DTPP based small molecules showed p-type behavior with hole mobility around 2.2 × 10^−4^ cm^2^ V^−1^ s^−1^ (Figure 2H). The low mobility could be ascribed to the simple structures, in which the DTPP core is flanked with a benzene ring. To further optimize these molecules, other donor units, such as thiophene, thienothiophene, and so on, instead of the benzene ring, could be used.

## Conclusion and Outlook

Until now, DPP chromophore and its derivatives, as one of the most popular acceptor units, were widely used in construction donor-acceptor materials, which showed high charge transfer mobility in the OFETs. However, the isoDPP-based ones received less attention though they are promising. In this article, DPP-based materials, including DPP, isoDPP, and DPP-based derivatives, as the semiconductor in OFETs were reviewed. Though DPP-based materials were promising in OFETs, great opportunities and challenges still remain in the development of DPP-based semiconductors with high charge carrier mobility, typically for the ambipolar type OFETs. To obtain high-performance DPP-based semiconductor materials, the highlighted development comes from the following factors: planar materials backbone, large π-conjugation system, highly crystalline, strong π-π stacking and aggregation, short molecular distance, and so on. Recently research reversed the idea that DPP chromophore is a promising unit to build high-performance semiconductor materials. Further optimizing its structures, typically the isoDPP-based materials, are always challenging and urgently necessary.

## Author Contributions

XZ and SC prepared the manuscript. JL revised the manuscript. XW and MZ supervised the whole work. All authors discussed and commented on the paper.

## Conflict of Interest

JL and MZ who were employed by company Qingdao Haiwan Science and Technology Industry Research Institute Co., Ltd. The remaining authors declare that the research was conducted in the absence of any commercial or financial relationships that could be construed as a potential conflict of interest.
